# Exploring Shank Circumference by Stretching after Training among Volleyball Players

**DOI:** 10.3390/ijerph18168849

**Published:** 2021-08-22

**Authors:** Yi-Lang Chen, Fang-Min Tsai, Wei-Chen Hsu, Chun-Ju Yang, Ting-Yo Yei

**Affiliations:** Department of Industrial Engineering and Management, Ming Chi University of Technology, New Taipei 24301, Taiwan; m09218014@mail2.mcut.edu.tw (F.-M.T.); m09218003@mail2.mcut.edu.tw (W.-C.H.); m09218009@mail2.mcut.edu.tw (C.-J.Y.); m09218010@mail2.mcut.edu.tw (T.-Y.Y.)

**Keywords:** stretching, shank circumference reduction, intervention time, subjective discomfort

## Abstract

This preliminary study examined the effects of a stretching intervention after training and its duration (15 vs. 30 min) on participants’ shank circumference (SC) reduction and subjective discomfort score. Ten male volleyball players underwent a routine 3 h training. A two-way analysis of variance revealed that the stretching intervention had significant effects on SC reduction (*p* < 0.01) and subjective discomfort scores (*p* < 0.001). Stretching after training could help eliminate shank strain, and a slighter discomfort in shanks when stretching was also seen (score, 20.1/100). An independent-samples *t* test revealed a significantly higher SC reduction (*p* < 0.01) with 30 min of stretching (5.6 mm) than with 15 min of stretching (2.7 mm); both stretching durations reduced SC significantly more than the no-stretching condition did. The findings of this study can serve as a reference for volleyball players to alleviate shank strain after daily routine training.

## 1. Introduction

Many athletes experience muscle soreness after intense exercise. Intensive muscle contractions (e.g., eccentric exercise) cause damage to muscle fibers [1] and may result in delayed-onset muscle soreness (DOMS) [2,3,4]. Many interventions, including stretching, massage, cryotherapy, laser therapy, nutritional strategies, proper sleep, ultrasound, pharmacological agents, and cool water immersion [3,5,6,7,8,9], have been used to prevent these symptoms. However, which recovery modalities are most effective is still questionable, especially with regard to the varying features of individual sports and athletes [9,10].

Volleyball consists of periods of low intensity followed by short bouts of high-intensity force application [11]. Players must dive, abruptly jump repeatedly, and change direction quickly, all of which involve intense eccentric muscle contractions. This intense muscular work can cause fatigue and thus affect players’ performance [7]. The recovery modalities utilized after volleyball competition or training thus become important in preventing injury, especially when athletes have little time between intensive trainings or even matches. Even though volleyball is popular worldwide, little research has examined recovery strategies for volleyball players [12].

A systematic literature review by Closs et al. [13] highlighted the specific methods used to recover after playing volleyball and evaluated their effectiveness. They found that some methods, such as nutrition and sleep regimens, have been definitively shown to benefit volleyball players, whereas others, such as cold water immersion and laser therapy, have shown promise but require further study to determine their overall effect. Unfortunately, the strategy of stretching for recovery was not included among these methods because very few published studies have been conducted on stretching recovery in volleyball players. Nevertheless, Barnett [5] surveyed the recovery modalities used between training sessions by elite athletes and speculated that the majority of studies assessing exercise-induced muscle injury and DOMS have used untrained participants performing unfamiliar eccentric exercise. Thus, the results may be unlikely to reflect the actual situations of these athletes.

Some recovery techniques, such as nutrition and sleep modalities, should be monitored by athletes themselves on an individual level, and their condition and response to recovery may not be significant until days or weeks later [13]. However, some studies examining the efficacy of recovery techniques have focused on exercise-induced muscle damage (e.g., DOMS) and pain or discomfort 1–2 days after exercising; one recovery technique is stretching [14,15,16,17]. Stretching has long been a common modality before and after training during different exercise stages [18]. The efficacy of stretching as an aid to performance is less apparent; however, the benefit may lie in the alleviation of discomfort and pain [16,19]. Particularly, chronic stretching may reduce injury [18].

The primary perceived function of stretching has been to increase the range of motion of the joints and to reduce musculotendinous stiffness to prevent injury and promote recovery [16,20,21,22]. For example, in French professional soccer, 50% of teams currently use stretching as a recovery strategy [15]. Although a mechanism by which stretching may enhance the recovery process has yet to be identified, stretching may disperse the swelling accumulated during tissue damage [23]. To assess the degree of swelling, studies [24,25,26] have employed an indicator to measure changes in shank circumference (SC). Increased SC indicates that leg strain is exacerbated because of increased accumulation of blood in the lower limbs due to restricted circulation [27], particularly within the perimuscular connective tissue and regions of the myotendinous junction, because of eccentric exercise [4,28]. Such swelling is characteristic of an acute inflammatory response to muscle damage or injury [29].

Because the recovery technique of stretching has been rarely evaluated in professional volleyball players, this study recruited 10 participants from a university volleyball team in Taiwan to examine the recovery effect of stretching on alleviating leg strain after regular daily training. Independent variables were stretching exercises (stretching vs. nonstretching) and time periods of stretching (15 vs. 30 min). SC reductions and subjective discomfort scores were collected in varying combinations as dependent variables to examine the effect of stretching on leg strain alleviation in volleyball players.

## 2. Materials and Methods

### 2.1. Participants

This study recruited 10 healthy male participants, all of whom were active volleyball players on a university team who were recommended for admission on the basis of their volleyball skill. The team qualifies for the National College Cup finals in Taiwan almost every year. The mean (standard deviation (SD)) age, height, body weight, and body mass index of the participants were 23.5 (1.6) y, 185.4 (4.7) cm, 78.8 (5.1) kg, and 22.9 (1.5) kg/m2, respectively. All participants were spikers; one participant jumps with his right foot and all others jump on their left feet. SC measurement was based on the jumping foot. Although the participants have experienced muscle soreness because of regular training or competition, the soreness was all reasonable as a postexercise effect. In the six months before the experiment, none of the participants sought any medical attention due to pain or injury. Prior to the test, all participants were informed of the experimental procedure of this study. The experiment was performed in accordance with the principles of the Declaration of Helsinki [30], and the Ethics Committee of National Taiwan University approved the experimental procedures. All study participants provided written consent before the experiment and received remuneration for participation.

### 2.2. Stretching Protocols

**Figure 1:**
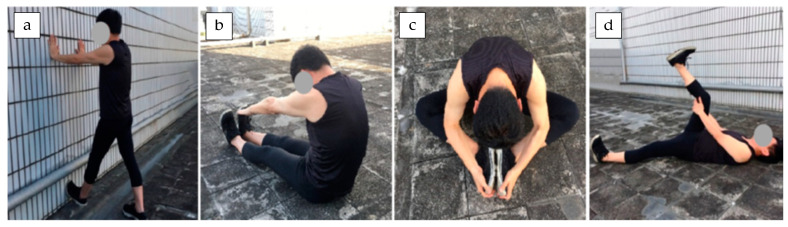
Four stretching protocols (**a**–**d**) after volleyball training used in this study.

The stretching exercises adopted in this study flexed and extended various body parts, particularly the legs. Four stretching protocols, which mainly included standing, sitting, and lying hamstring stretches [31,32,33,34], were implemented with reference to the long-term practice of the university volleyball team:

1. Standing stretching: Participants stood with their hands against a wall, then alternately lifted their right and left toes against the wall to stretch their hamstrings (Figure 1a).
2. Sitting stretch (I): Participants sat on the floor and kept their legs straight, then bent their trunk forward and reached their hands as close as possible to their toes (Figure 1b).
3. Sitting stretch (II): Participants sat on the floor and flexed their legs such that their knees were close to the ground; they used their hands to help make the soles of their feet touch completely flat against each other. Participants bent their trunk forward as far as possible (Figure 1c).
4. Lying-down stretching: Participants laid on the floor and lifted a single leg, using their hands to bend the leg toward their face as far as possible. They then switched legs (Figure 1d).

During stretching, each of the 4 protocols were performed over 1 min and repeated until the participants were instructed to stop stretching. Stretching frequency was controlled using a digital metronome for a total bout of stretching of 15 s (stretching followed by resting, 7.5 s of each) for trunk flexion, hamstring extension, or leg/foot lifting. The 4 stretching bouts were performed by participants over 1 min and repeated.

### 2.3. SC Measurement

On the basis of the SC measurements proposed in previous studies [24,25], we used a pull-push tester (MP-1; Attonic, Aichi, Japan) to control the fixed force applied during the measurement to minimize errors. We measured the participants’ SC at the midpoint between the knee and ankle joints (the point was marked during the whole testing period). During SC measurement, participants were asked to stand on the ground, and each SC measurement was repeated three times. The average of the two closest values was then used for further analyses.

### 2.4. Visual Analog Scale for Discomfort Rating

In this study, subjective assessments of lower limb discomfort were performed using a continuous visual analog scale (VAS) [35]. The VAS has been reported to be a reliable assessment of perception and more precise than an ordinal scale that ranks responses; the VAS used in the present study was modeled after the comfort scales developed by Mündermann et al. [36]. The left end of the scale was labeled “No discomfort at all”, and the right end was labeled “Extreme discomfort”. The participants used a pen to complete ratings by marking locations along the scale that most accurately represented their feeling of discomfort. An experimenter used a ruler to measure the distance from the “No discomfort at all” anchor to the location of a mark, and this distance was used for analysis.

### 2.5. Experimental Design and Procedure

**Figure 2:**
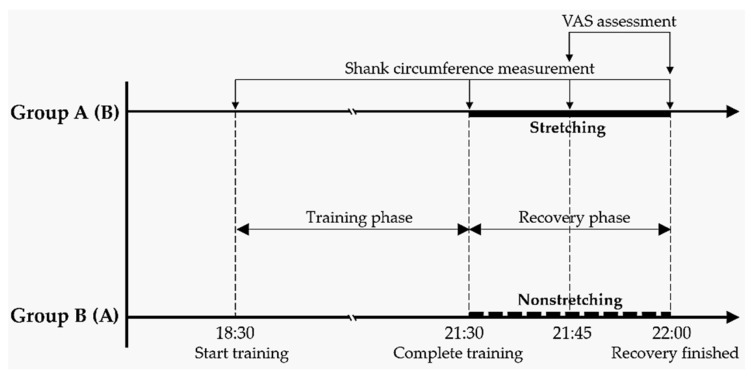
Schematic timeline of study design (VAS, visual analog scale).

This study collected SC values and subjective discomfort scores for the lower limbs with two stretching conditions (with and without stretching) after training and two stretching or no-stretching durations (15 and 30 min; SC and VAS values were collected at these two time points). The ten participants were randomly divided into two groups (groups A and B) with five participants each (Figure 2). After regular daily training from 18:30 to 21:30, the participants in groups A and B were asked to perform stretching and non-stretching trials, respectively. The SC values were collected for each group before and after training (0 min) and the subsequent stretching or resting period at 15 and 30 min. The VAS measurements were reported by participants only after 15 and 30 min. The participants in group B (resting group) were requested to sit on the floor without any effort. On the next day, the two groups were alternated. The process was repeated twice, and the average value in SC of each participant was used for further analyses. All SC reductions were calculated on the basis of data measurements of each participant.

### 2.6. Statistical Analysis

The data collected in the study were analyzed using SPSS Statistics 23.0 (IBM Corp., Armonk, NY, USA), and a significance level of α = 0.05 was used for all tests. Data collected from the participants were analyzed using descriptive statistics (i.e., mean, SD, and independent-samples *t* test). Two-way analysis of variance (ANOVA) was used to examine the effects of the two stretching conditions (with and without stretching) and the two time periods (15 and 30 min) on SC reduction and VAS values. However, the SC reduction was calculated on the basis of SC data recorded when training was completed.

## 3. Results

**Figure 3:**
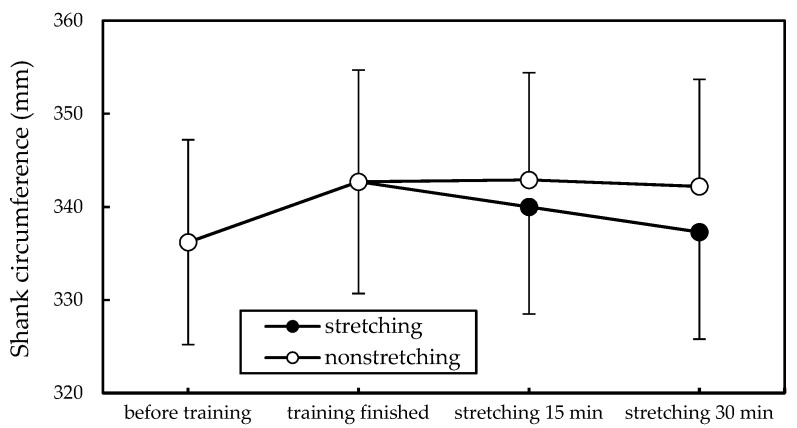
Comparison of shank circumferences (mean with standard deviation) at different measurement stages.

The two-way ANOVA revealed that both stretching (*p* < 0.01) and stretching duration (*p* < 0.05) significantly reduced SC (Table 1), and stretching for 30 min was associated with a high SC reduction (Figure 3). Subjective discomfort scores were significantly influenced by stretching (*p* < 0.001) but not by stretching duration. The overall mean (SD) discomfort score reported by the stretching group (20.1 [22.0]) was higher than that reported by the no-stretching group (7.8 [10.0]).

Table 2 shows the *t* test results of various test conditions in pairs. When stretching was performed, the effect of stretching for 30 min on participants’ SC reduction significantly increased compared with the effect of stretching for 15 min (*p* < 0.01), with a difference of 2.9 mm. The SC reduction in the no-stretching condition was also higher at 30 min than at 15 min; however, this finding was not significant. Regardless of duration, SC reduction with stretching was significantly higher than without stretching.

## 4. Discussion

This study is the first to examine the effect of stretching on alleviating lower extremity strain after volleyball training. Reduction in SC was considered an index of eliminated strain. The results show that stretching after training had benefits for SC reduction and that the duration also affected the degree of SC reduction. Stretching for a longer time (30 min in this study) could lead to better SC reduction.

Figure 3 illustrates the alterations of SC at various measurement points. The SC of participants increased from 336.2 mm before training to 342.7 mm after training for 3 h. However, the non-stretching SC values hardly changed within 30 min after training, whereas stretching reduced the SC to 340.0 mm and 337.3 mm at 15 and 30 min, respectively. The SC recovered and was slightly greater than before training, with a difference of 1.1 mm. The SD in Figure 3 appeared high because of the individual differences in SC values. All SC reductions were calculated on the basis of measurements of each participant to minimize the effect of the variation between individuals.

Bobbert et al. [23] proposed that the static stretching of sore muscles after exercise can force the dispersion of swelling that accumulates after tissue damage. Repeated stretching reduces tension on the muscle-tendon unit at any given length. Stretching may be the most effective means of alleviating pain during DOMS. Because the jumping attack in volleyball is the primary source of strain on an athlete’s body, especially the legs, we adopted SC as an indicator of leg strain (swelling) [24,26]. Stretching has been demonstrated to have no effect on the alleviation of muscle soreness or other DOMS symptoms in previous studies. Cheung et al. [3] stated that one explanation is that studies employing stretches of less than 30 s may be limited by the stretch reflex response. Only minimal stretching lasting 0 to 5 min has been generally reported in stretching studies [3]. Stone et al. [18] also indicated that acute stretching has little effect on injury, whereas chronic stretching may. The Premier League reported a mean static stretching holding time of approximately 30 s and a mean number of repetitions per hamstring muscle group of 3 per session. In French professional soccer, even though 50% of teams currently use stretching as a recovery strategy [15], no substantial scientific evidence has supported the use of stretching to enhance the postexercise recovery of soccer players [37]. In the present study, stretching was followed immediately by volleyball training and implemented continuously for longer periods, and SC reductions were examined at the middle (15 min) and final (30 min) points of the whole period.

Our findings also reveal that the subjective discomfort score with no stretching (i.e., seated resting) was significantly lower than that with stretching (7.8 vs. 20.1). However, the VAS score is designated on a scale from 0 to 100 mm with the anchors of 0 for “No discomfort at all” to 100 for “Extreme discomfort”. In other words, a score of 20 was a slightly uncomfortable level [36]. Compared with the effect on SC reduction, stretching after training is still of practical value.

Recovery techniques after volleyball training tend to have long-term effects (nutritional strategies, proper sleep, mental and psychological techniques) and some short-term effects (cold water immersion and laser therapy), but no relevant research has been conducted on stretching [13]. Herbert et al. [38] reported that stretching was not clinically worthwhile for reducing muscle soreness in the days after exercise; however, they did not perform an immediate intervention after training. Studies have also found that on the basis of differences in physical characteristics, the recovery effects between untrained people and professional athletes vary [5,15]. The four stretching protocols adopted in the present study were developed and modified based on stretching techniques used for years by the university volleyball team. Some volleyball team members had questioned the effect of stretching, which motivated us to conduct the study.

This study has several limitations. First, it was limited by the qualifications of the participants. Only 10 active attackers from the university volleyball team were recruited; this sample size is limited to a single team and is relatively small. A multi-site study would allow an increase in subject numbers for future research. In addition, this study was a cross-sectional study that extended the findings for the DOMS effect or fatigue recovery, but further investigation is needed. SC reduction was used as an indicator of leg strain; whether its representativeness was sufficient requires more examination for clarification.

## 5. Conclusions

This preliminary study recruited 10 university volleyball players to examine the effect of stretching on SC reduction after training. The results showed that stretching had a significant effect on reducing SC. Although stretching may cause some discomfort, SC reduction differed significantly between 15 min of stretching and no stretching, and SC reduction was optimal with stretching for 30 min.

## Figures and Tables

**Table 1:** Two-way analysis of variance results.

Responses	Variables	*df*	MS	*F*	Significance	Power
Shank circumference reduction	Stretching (S)	1	93.52	8.74	*p* < 0.01	0.792
	Time period (T)	1	59.48	4.62	*p* < 0.05	0.747
	S × T	1	12.07	0.95	*p* = 0.333	0.161
Discomfort score	Stretching (S)	1	2919.96	10.04	*p* < 0.001	0.878
	Time period (T)	1	231.50	0.80	*p* = 0.375	0.142
	S × T	1	48.54	0.17	*p* = 0.684	0.069

**Table 2:** Shank circumference reduction between various testing combinations (in mm).

Levels	Stretching	No-Stretching	*t* Test Results
15 min	2.7 (1.4)	−0.2 (1.0)	*t* = 5.839, *p* < 0.001
30 min	5.6 (2.1)	0.5 (1.7)	*t* = 6.487, *p* < 0.001
*t* test results	*t* = −4.525, *p* < 0.01	*t* = 1.350, *p* = 0.195	

## Data Availability

The data presented in this study are available on request from the corresponding author. The data are not publicly available due to privacy reasons.
